# Copy Number Analysis Reveal Genetic Risks of Penile Cancer

**DOI:** 10.3389/fonc.2020.596261

**Published:** 2020-12-14

**Authors:** Yongbo Yu, Chengwen Gao, Yuanbin Chen, Meilan Wang, Jianfeng Zhang, Xiaocheng Ma, Shuaihong Liu, Hang Yuan, Zhiqiang Li, Haitao Niu

**Affiliations:** ^1^ Urology Department, The Affiliated Hospital of Qingdao University, Qingdao, China; ^2^ Laboratory of Medical Biology, Medical Research Center, The Affiliated Hospital of Qingdao University & The Biomedical Sciences Institute of Qingdao University (Qingdao Branch of SJTU Bio-X Institutes), Qingdao University, Qingdao, China; ^3^ Nursing Department, The Shengli College, China University of Petroleum, Dongying, China

**Keywords:** penile cancer, copy number alterations, *MYCN*, *FAK*, *TP53*

## Abstract

**Objectives:**

To evaluate copy number alterations (CNAs) in genes associated with penile cancer (PeC) and determine their correlation and prognostic ability with PeC.

**Methods:**

Whole-exome sequencing was performed for tumor tissue and matched normal DNA of 35 patients diagnosed with penile squamous cell carcinoma from 2011 to 2016. Somatic CNAs were detected using the Genome Analysis Toolkit (GATK). Retrospective clinical data were collected and analyzed. All the data were statistically analyzed using SPSS 16.0 software. The cancer-specific survival rates were estimated by Kaplan-Meier curves and compared with the log-rank test.

**Results:**

CNAs in the *MYCN* gene was detected in 19 (amplification: 54.29%) patients. Other CNAs gene targets were *FAK* (amplification: 45.72%, deletion: 8.57%), *TP53* (amplification: 2.86%, deletion: 51.43%), *TRKA* (amplification: 34.29%, deletion: 2.86%), *p75NTR* (amplification: 5.71%, deletion: 42.86%), *Miz-1* (amplification: 14.29%, deletion: 20.00%), *Max* (amplification: 17.14%, deletion: 2.86%), *Bmi1* (amplification:14.29%, deletion: 48.57%), and *MDM2* (amplification: 5.71%, deletion: 45.72%). The CNAs in *MYCN* and *FAK* correlated significantly with patient prognosis (P<0.05). The 3-year Recurrence-free survival rate was 87.10% among patients followed up. The 5-year survival rate of patients with *MYCN* amplification was 69.2%, compared to 94.4% in the non-amplification group. The 5-year survival rate of patients with *FAK* amplification was 65.6%, compared to 94.7% in the non-amplification group. The PPI network showed that *TP53* and *MYCN* might play meaningful functional roles in PeC.

**Conclusion:**

*MYCN* and *FAK* amplification and *TP53* deletion were apparent in PeC. *MYCN* and *TP53* were hub genes in PeC. *MYCN* and *FAK* amplification was also detected and analyzed, and the findings indicated that these two genes are predictors of poor prognosis in PeC.

## Introduction

Penile cancer (PeC) is a rare and aggressive malignant tumor that accounts for less than 1% of carcinomas in males in the United States (1). The regional differences in incidence are significant, with the high incidence in the developing countries (2.8–6.8 per 100,000), where the low rate of neonatal circumcision and socioeconomic conditions make the patients vulnerable to a variety of risk factors. Various have been identified, including lack of circumcision, phimosis, smoking, balanitis, obesity, lichen sclerosus, and psoralen UV-A phototherapy, contributing to the courses of PeC. Moreover, human papilloma virus (HPV) has been linked to nearly 40%–50% of cases (2), and the molecular mechanism. Several studies have detected somatic changes that arise in (3–5), but few of them were based on whole-exome sequencing (5). Our previous study has performed a whole-exome sequencing analysis of PeC, and identified recurrent mutations in 11 genes (6).

Copy number alterations (CNAs) are somatic changes that cause the amplification or deletion of DNA fragment (7, 8), and represent the most common alterations of cancer cells (7, 9, 10). They contribute to both onset and progression of cancer by inappropriate activation of proto-oncogenes and/or inactivation of tumor suppressor genes (11, 12). Similar to genome-wide association studies (GWAS) that help find single nucleotide polymorphisms associated with disease phenotypes, GWAS can be extended to CNAs to help find structural variations associated with human traits and diseases (13). To date, the CNAs of PIK3CA, IL-22 and MYC have been reported in PeC. PIK3CA copy number amplification was found to have no prognostic value for cancer-specific survival (14). The function of IL-22 copy number amplifications in PeC is not clear (15). However, MYC amplification increases during PeC progression (16–18).

*MYCN*, an MYC family member, is a proto-oncogene that is mainly expressed in primarily neuronal cell lineages during embryogenesis and could be involved in tumorigenesis when uninhibited (19). The *MYCN* oncogene is amplified in approximately 20% of neuroblastomas (NBs) (20). *MYCN* belongs to the Myc/Max/Mad/Mnt network of proteins that regulate proliferation, apoptosis, and differentiation (21), which were amplified in neuroblastoma, small-cell lung cancer (22) and hepatic cancer (23), respectively.

Because knowledge of the pathogenesis and carcinogenesis of PeC remains limited. In the present study, we performed a whole-exome sequencing analysis of PeC in Han Chinese patients to search for the relationship between CNAs and clinical characters in penile cancer.

## Materials and Methods

### Sample Source and Clinical Characteristics

Fresh PeC and blood samples from 35 PeC patients diagnosed from 2011 to 2016 were collected in the Affiliated Hospital of Qingdao University and frozen at constant temperature of −80°C. The patients were followed up by telephone and outpatient service to assess their health. All the tumor samples used for DNA extraction and exome sequencing were confirmed to have 80% tumor content by an experienced pathologist. PSCC diagnoses were made according to the clinical history, physical examination, and biopsy results. Primary treatment for PSCC included partial or radical penectomy with concomitant inguinal lymph node dissection (ILD), which included ipsilateral or ilioinguinal lymphadenectomy *via* contralateral superficial inguinal or ilioinguinal dissection according to the clinical condition. Recurrence-free survival was defined as the period from the time of present surgery in our hospital to tumor recurrence (or last follow-up visit).

Clinical information for 35 patients, including gender, age, patient number, sample acquisition method, tumor size, pathological subtype, differentiated degree, local infiltration status, WHO grade, and follow-up results (recurrence and survival), was collected and summarized in **Table S1**. Informed consent was obtained from all human subjects, and our study was approved by the Affiliated Hospital of Qingdao University Ethics Committee.

### Whole-Exome Sequencing

In order to capture the tumor and blood exonic region, library preparation was performed with Agilent SureSelect Human All Exon V5+UTR Kits (Santa Clara, CA) according to the manufacturer’s guidelines. Samples were deeply sequenced on the Illumina HiSeq2500 platform. The mean per-target depth of coverage across all targets was 92, with 89% of targets sequenced to an average of 10× or greater.

### CNAs Detection

Raw reads from each library were quality controlled with FastQC, trimmed using Trimmomatic and then mapped to the reference human genome (NCBI build 38, hg 38) by a Burrows Wheeler Alignment (BWA) tool v 0.7.17 (24) with the BWA-maximal exact match algorithm and default parameters. PCR duplicates were flagged with Picard, and the outputs were locally realigned using the Indel Realignment tool of the Genome Analysis Toolkit (GATK) version 4.1.2.0 (25). After local realignment, the BaseRecalibrator tool from GATK was used for recalibration. CNAs in normal samples were compared to matching tumor samples using a relative coverage method performed in GATK. GISTIC version 2.0 (http://archive.broadinstitute.org/cancer/cga/gistic) analysis was performed to identify significantly recurrent copy number amplification and deletion at focal level. A log2 ratio above 0.1 was considered as “amplification,” and a log2 ratio below -0.1 was considered as “deletion.” (https://gatkforums.broadinstitute.org/firecloud/discussion/8254/gistic2-0)

### KEGG Pathway Analysis

The Kyoto Encyclopedia of Genes and Genomes (KEGG) pathway database can be seen as a set of orthologue group tables including category pathways, subcategory pathways and secondary pathways, which are often encoded by positionally coupled genes on the chromosome and much meaningful in predicting gene functions (3). To analyze the possible relationship between related genes and PeC, we characterized somatic mutations of this pathway for PeC, which was absent in KEGG. One canonical signaling pathway, *MYCN*/Max, was found to be altered at varying frequencies in the different cancer types analyzed by KEGG (https://www.kegg.jp/kegg-bin/highlight_pathway?scale=1.0&map=map05202&keyword=MYCN).

### Analysis of the PPI Network

The STRING database (http://string-db.org/) is a precomputed global resource for predicting functional associations between proteins. In this paper, the STRING online tool was applied to analyze the protein-protein interactions (PPIs) of the CNA genes in PeC with the threshold of combined score >0.4.

### Statistical Analysis

All the data collected were statistically analyzed by SPSS 16.0 software. We divided the patients into two groups based on the CNA results: the normal target gene group and the abnormal target gene amplification group. Patient status information was obtained through outpatient or telephone follow-up. Chi-square analysis and Kaplan-Meier survival curves were used to compare the five-year survival rates among different groups, and significant differences were determined using the log-rank test. All *P* values were two-sided, and a *P* value < 0.05 was considered to indicate statistical significance. Figures were edited with Origin 8 software.

## Results

### Clinical Characteristics and Gene Variations

The median age of the 35 patients participating in exome sequencing analysis was 63 years (range 27–86 years). Twenty-three patients (65.71%) had redundant prepuce, 9 patients (25.71%) had phimosis, and nobody had been circumcised. All patients were diagnosed with penile squamous cell carcinoma. The majority of primary lesions presented on the corpus cavernosum (n = 16; 45.71%) and occasionally involved the adjacent structures (submucosa [n = 5; 14.29%], dartos [n = 1; 2.86%], subcutaneous soft tissue [n = 1; 2.86%]), and 5 specimens could not be defined. Sixteen (45.71%) patients were diagnosed with pT stage expressing T2 disease. The proportion of histologically well to high and moderately differentiated cases were 57.14% and 31.43%, separately. The proportion of samples with low differentiation was 8.82%, and 1 case was unknown. Partial penectomy was performed in the majority of cases (n = 29; 82.86%). Five patients also underwent inguinal lymph node dissection in addition to surgical removal of the lesion, and the result showed that 2 cases were positive. The median follow-up time was 60 months, and the number of patients lost to follow-up was 4. Recurrence occurred in four patients within 3 years after surgery and The 3-year Recurrence-free survival rate was 87.10%. The overall 3- and 5-year cancer-specific survival (CSS) rates were 87.10% and 83.87%, respectively.

For HPV detection, only 30 samples were tested for HPV and five were not. Among 30 PSCC samples, six were found to be HPV-positive. Moreover, ﬁve were HPV type 16 and one appeared HPV types 18 and 81. Clinicopathologic data were obtained from electronic clinical medical records and are shown in Supporting Information **Table S1**.

**Figure 1:**
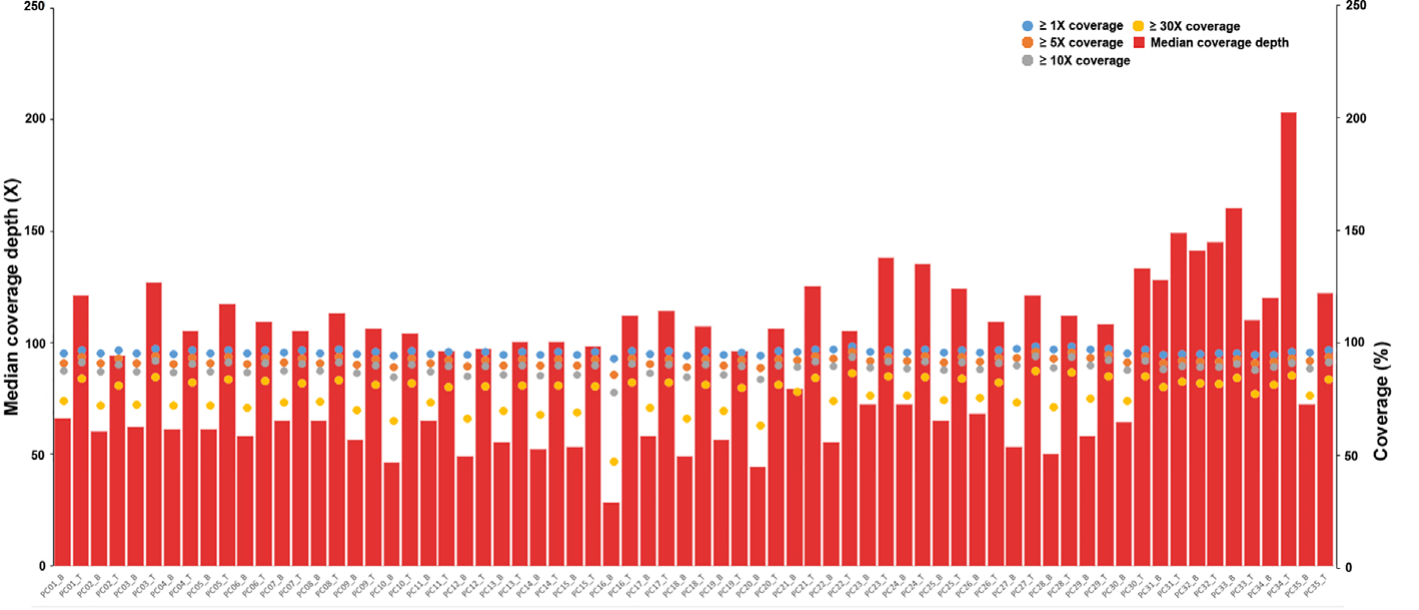
Depth and coverage of 35 paired samples. The histograms represent the average sequencing depth of each sample, and the specific values are shown on the left axis. The scatter plot shows the distribution of all samples under coverage values of 1×, 5×, 10× and 30×, and the specific values refer to the right axis.

The average sequencing depth was 120× for the tumor samples and 70× for matched normal blood samples, with ≥10× coverage for 89.0% of the target regions in tumor samples and 88.7% of the target regions in blood samples (**Figure 1** and **Table S1**).

### Mutational Features and Pathway Alterations Relative to PeC

**Figure 2:**
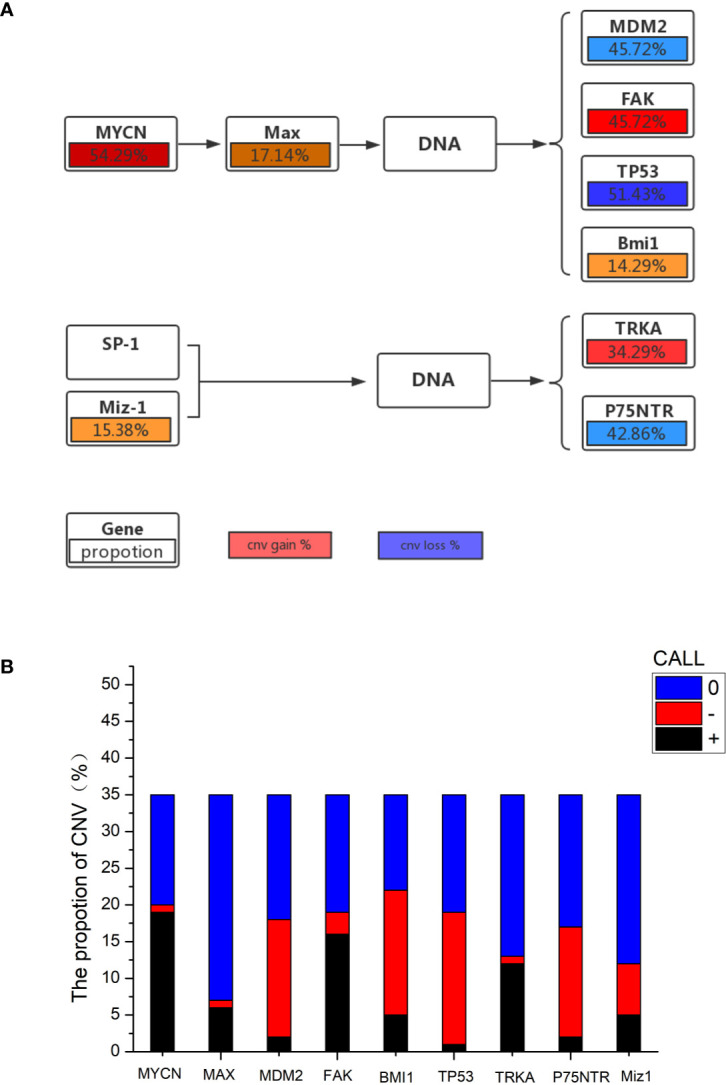
**(A)** Proportion of gene copy number variation in the KEGG pathway. **(B)** Proportion of gene copy number variation: 0: normal gene number; –: gene copy number deletion. +: gene copy number amplification.

The mutational features of the PeC sample were also assessed. These features can be indicative of specific mutagenic mechanisms promoting tumorigenesis. A gene set analysis was performed with KEGG to determine pathways associated with PeC. In our PeC samples, this pathway had at least one alteration, and most alterations were found in 19 tumors (54.29% of samples). The relative pathway targets involve 10 kinds of proteins (**Figure 2A**). Nine target proteins were detected in PeC samples, except for sp-1 in the known pathway. Most mutations caused amplification of the affected genes, including *MYCN, Max, FAK, SP-1* and *TRKA*, while *MDM2, Bmi1,TP53*, *p75NTR*, and *Miz-1were more likely to* exhibits downregulation levels.

The copy number amplification of *MYCN* was 19 (54.29%), which is the largest number of mutations in the analysis. The remaining targets were *FAK* (amplification: 45.72%, deletion: 8.57%), *TP53* (amplification: 2.86%, deletion: 51.43%), *TRKA* (amplification: 34.29%, deletion: 2.86%), *p75NTR* (amplification: 5.71%, deletion: 42.86%), *Miz-1* (amplification: 14.29%, deletion: 20.00%), *Max* (amplification: 17.14%, deletion: 2.86%), *Bmi1*(amplification:14.29%, deletion: 48.57%), and *MDM2* (amplification: 5.71%, deletion: 45.72%). Therein, the *TP53* and *p75NTR* mutations were inactivating, and the remaining targets were amplified. Detailed information about the samples for the exome sequencing analysis can be found in Supporting Information **Table 1** and **Figure 2B**.

**Table 1:** Proportion of gene copy number alterations.

Gene	CNA	Number	Total	Proportion	Variation Proportion
*MYCN*	+	19	35	54.29%	57.14%
	−	1		2.86%	
	0	15		42.86%	
*Max*	+	6	35	17.14%	20.00%
	−	1		2.86%	
	0	28		80.00%	
*MDM2*	+	2	35	5.71%	51.43%
	−	16		45.72%	
	0	17		48.57%	
*PTK2(FAK*)	+	16	35	45.72%	54.29%
	−	3		8.57%	
	0	16		45.72%	
*Bmi1*	+	5	35	14.29%	62.86%
	−	17		48.57%	
	0	13		37.14%	
*TP53*	+	1	35	2.86%	54.29%
	–	18		51.43%	
	0	16		45.71%	
*NTRK1(TRKA*)	+	12	35	34.29%	37.14%
	−	1		2.86%	
	0	22		62.86%	
*NGFR(p75NTR*)	+	2	35	5.71%	48.57%
	−	15		42.86%	
	0	18		51.43%	
*ZBTB17(M*iz1)	+	5	35	14.29%	34.29%
	−	7		20.00%	
	0	23		65.71%	

### Correlation and Prognosis Between CNAs and PeC

**Figure 3:**
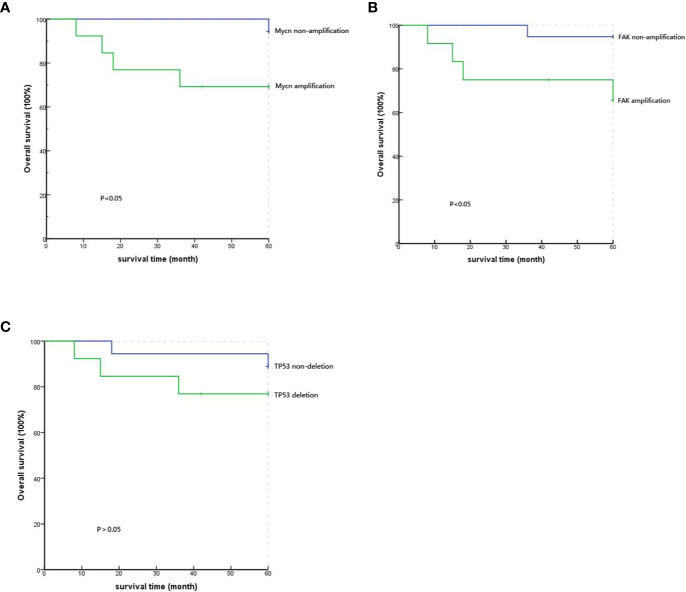
Comparison of the 5-year survival rate with different gene variations by Kaplan-Meier analysis. **(A)** Comparison of the 5-year survival rate between *MYCN* amplification and *MYCN* non-amplification showed significant difference (P=0.047). **(B)** Comparison of the 5-year survival rate between *FAK* amplification and *FAK* non-amplification showed a significant difference (P=0.032). **(C)** Comparison of the 5-year survival rate between *TP53* deletion and *TP53* nondeletion showed no significant difference (P=0.329).

The 35 patients’ follow-up information was obtained through outpatient and telephone visits. The rate of lost follow-up was 11.4%, and four patients could not be contacted. Finally, only 31 patients were included to analyze the correlation between prognosis and gene mutations. According to the results of the Kaplan-Meier survival curve, the five-year survival rates of the *MYCN* amplification and *MYCN* non-amplification groups were 69.2% and 94.4%, respectively. Simultaneously, the P value from the log-rank test was 0.047<0.05, which means that the difference between the groups with significantly correlation and *MYCN* was an independent prognostic factor of PeC (**Figure 3A**). Analogously, the five-year survival rates of the *FAK* non-amplification group and the *FAK* amplification group were 94.7% and 65.6%, respectively. The P value from the log-rank test was 0.032<0.05, which means that the difference between the groups was statistically significant, and *FAK* amplification could act as an independent prognostic factor of PeC (**Figure 3B**). However, the five-year survival rates of the normal *TP53* group and *TP53* inactivation group were 88.9% and 76.9%, respectively. The P value from the log-rank test was 0.329<0.05, which means that the difference between the groups without correlation and *TP53* inactivation was not the prognostic value of PeC (**Figure 3C**).

### PPI Network Constructed for CNA Genes in PeC

**Figure 4:**
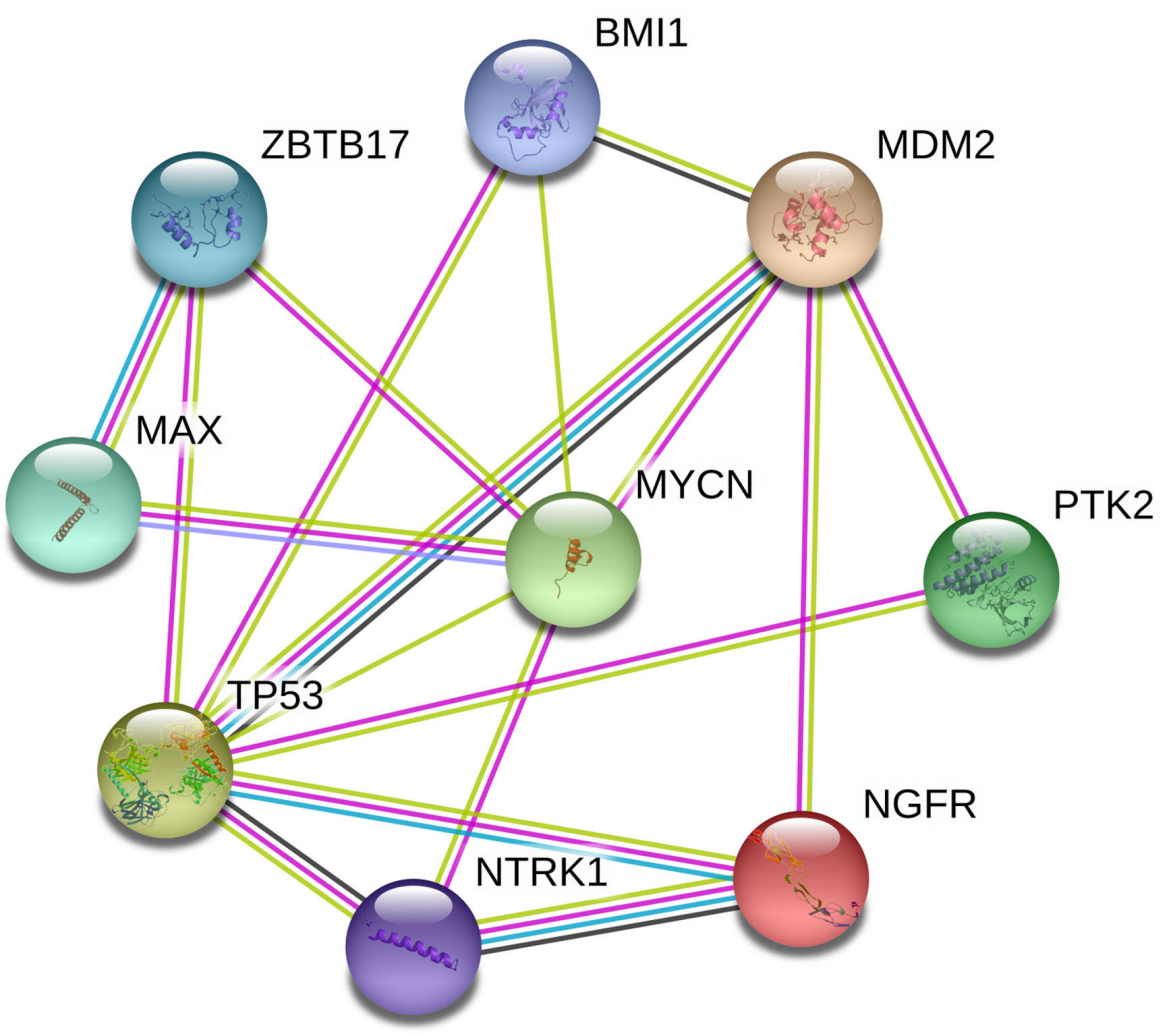
Protein-protein interaction network of copy number alteration (CNA) genes in penile cancer (PeC). Circles represent genes, lines represent the interaction of proteins encoded by the genes, and the results within the circle represent the structures of the proteins. The line color represents evidence of the interaction between the proteins.

We constructed a PPI network of proteins encoded by CNA genes in PeC based on the PPI network, and the present study identified the top 2 hub genes: *TP53* and *MYCN* (**Figure 4**). These two genes might play meaningful functional roles in PeC.

## Discussion

PeC is a rare malignancy in the developed world but is much more common in developing countries. The genetic and molecular basis of PeC is still poorly understood (5), and further understanding of these aspects is important to improve our ability to diagnose, treat and prevent PeC. In our previous study (6), we characterized the PSCC genomic landscape using whole-exome sequencing. Of the 30 paired blood and tumor samples, recurrent mutations were identified in 11 genes; we also observed the frequently altered pathways for potential. The present study was based on the previous study in the samples and another five samples was added to the present study but not done HPV detection. SNPs have been reported in the previous study (6), so we’re not going to repeat it here.

Overall survival and recurrence-free survival in our study were higher than in Jong Won’ s study (26), the possible reason for which was that the pathological stage of the patients in our study were all≤T2, while the pathological stage ≥T3 accounts for 30.2% in Jong Won’ s study resulting to the lower OS and RFS. The patients in our study had earlier stages of pathology, perhaps because modern people in our region are more concerned about their health.

Currently, significant poor prognosticator in patients with penile cancer include lymph node positivity (27), metastatic nodes≥ 4 (28), AJCC stage ≥ III disease (26), pathologic stage of the primary tumor (29), histologic grade<G1 (30, 31), a tumor thickness≥ 5 mm (32), vertical growth pattern (33), age<53, Lactate Dehydrogenase (LDH)<188.5 U/L and Platelet-lymphocyte Ratio (PLR)<133.5 (34), p53 positivity (31), Human papillomavirus infection (35). However, there are few studies on the prognosis of penile cancer at the gene level, so our study also studied the gene types of prognostic factors later.

Our results provide the first detection of a *MYCN* CNA in the somatic mutant spectrum of PeC in Chinese men through whole-exome sequencing. In our sample, the *MYCN* amplification detection rate was 54.29%; *MYCN* is the type of gene in which we detected the highest mutation rate. In addition, the CNAs associated with *FAK* amplification and *TP53* deletion were found in more than one-third of the total samples tested. Furthermore, variations in other genes, such as *Max*, *Miz-1*, *Bmi1*, and *p75NTR*, were found in more than 10% of the samples.

The *MYCN* oncogene plays an important role in human tumorigenesis and has been proven to bind to gene promoters for various oncogenes involved in multiple life activities (36) and to increase the expression of many downstream targets. Previous data have indicated that the primary function of *MYCN* is as a transcription factor known to specifically bind the DNA E-box sequence CACGTG (37). A later study supports a dual role for *MYCN*. Murphy et al. showed that *MYCN* binds more often to the CATGTG E-box sequence, and *MYCN* binding is associated with DNA hypermethylation and can therefore also serve as an intermediary for chromatin structure-mediated regulation of various cellular processes, including cell growth, cell proliferation and cell differentiation (38). As a hub gene identified by our PPI study, *MYCN* plays an important regulatory role in its related pathways.

A previous study (39) found that *MYCN* amplification is one of the most significant prognostic indicators of neuroblastoma and is associated with rapid tumor progression and poor prognosis. Similarly, our data analysis found that *MYCN* was correlated with the prognosis of PeC, *MYCN* amplification indicated a poor prognosis. Lloveras B’s study (40) found that the *MYC* copy number amplification was significantly associated with poor outcome (mortality, node metastasis and/or recurrence) in PeC. As a member of the *MYC* family of proto-oncogenes, *MYCN* is more likely to appear as an independent prognostic indicator of PeC if the sample size is increased.

Studies (36) found that in neuroblastoma, the relationship between *MYCN* amplification and cell activity and aggressiveness suggests a potential relationship between focal adhesion kinase (*FAK*) and *MYCN*, since *FAK* is a key protein involved in cell activity.

*FAK* is a nonreceptor protein tyrosine kinase that targets focal adhesion and controls multiple cellular signaling pathways, including proliferation and survival (41). The inhibition of *FAK* activation has been found to affect a number of cellular pathways (36). *FAK* overexpression has also been shown in human sarcoma and melanoma tumors (42). A previous study (43) found that *MYCN* binds to the *FAK* promoter *in vitro* and *in vivo*, resulting in upregulation of *FAK* with electrophoretic mobility shift, chromatin immunoprecipitation (ChIP), and dual luciferase assays. Therefore, it is well proven that *MYCN*-regulated *FAK* intervention affects the prognosis of patients, which is consistent with our finding that *FAK* amplification could be an independent prognostic factor of PeC.

Cloning and evaluation of the *FAK* promoter has shown that it has many binding sites for various oncogenes, such as *TP53 (* 44). The tumor protein *TP53* (*TP53* or *TTP53*) was the first tumor suppressor gene, discovered in 1979, and is the guardian of the genome (45). *TP53* is the most widely studied tumor suppressor gene, playing an important role in inhibiting tumor development. The function of the *TP53* gene is to inhibit cell proliferation in response to DNA damage. By regulating target genes, *TP53* induces a variety of cellular responses, including growth arrest, senescence, and apoptosis (46, 47). However, the mutated *TP53* protein loses its protection against genomic functions, including the ability to inhibit cell proliferation and induce apoptosis, when mutated (48). Mutations in the *TP53* gene occur in most malignant tumors, such as lung cancer (49) and breast cancer (50). At the genetic level, carcinogenesis is a multistep process in which both oncogene activation and tumor suppressor gene inactivation are involved. Examination of the samples revealed a large number of SNP mutations in *TP53* in PeC, and the most common mutation classifications were missense mutation and nonsense mutation. These may account for the role of *TP53* in the molecular etiology of PeC.

In this study, a large number of gene variants of the samples were found in the *MYCN/Max* pathway in PeC, especially in *MYCN*. In addition, the *MYCN* and *FAK* CNA is associated with the prognosis of PeC, and its high expression level indicates a poor prognosis. However, mutations in *TP53* were not found to be related to prognosis, perhaps because our sample size was insufficient; it is necessary to carry out relevant tests with a larger sample size in the future.

## Data Availability Statement

According to national legislation/guidelines, specifically the Administrative Regulations of the People’s Republic of China on Human Genetic Resources (http://www.gov.cn/zhengce/content/2019-06/10/content_5398829.htm, http://english.www.gov.cn/policies/latest_releases/2019/06/10/content_281476708945462.htm), no additional raw data is available at this time. Data of this project can be accessed after an approval application to the China National Genebank (CNGB, https://db.cngb.org/cnsa/). Please refer to https://db.cngb.org/, or email: CNGBdb@cngb.org for detailed application guidance. The accession code CNP0001368 should be included in the application.

## Ethics Statement

The studies involving human participants were reviewed and approved by the Affiliated Hospital of Qingdao University Ethics Committee. The patients/participants provided their written informed consent to participate in this study. Written informed consent was obtained from the individual(s) for the publication of any potentially identifiable images or data included in this article.

## Author Contributions

YY, CG, and ZL contributed conception and design of the study. YY and YC organized the database. MW performed the statistical analysis. YY wrote the first draft of the manuscript. CG, YC, MW, and JZ wrote sections of the manuscript. XM, SL, and HY read and revised the manuscript. HN provided approval for publication of the content. All authors contributed to the article and approved the submitted version.

## Funding

This work was supported by the National Natural Science Foundation of China (82071750, 81772713, 81472411, 81372752, 81401899, 81871055, 81701321, 81421061, 31325014, 81130022, 21375139, 31571012 and 81501154), Taishan Scholar Program of Shandong Province (tsqn20161077), National Basic Research Program of China (973 Program; 2015CB559100), National Key R&D Program of China (2016YFC0903402, 2016YFC1306903, 2016YFC0902403, and 2017YFC0908105), Program of Shanghai Academic Research Leader (15XD1502200), Natural Science Foundation of Shandong Province (ZR2014HM088 and ZR2016HQ18), Major Science and technology innovation project of Shandong Province (2019JZZY021002), Key projects of Qingdao Science and Technology Program (18-6-1-64-nsh), Key Research and Development Program of Shandong Province (2018GSF118197), China Postdoctoral Science Foundation (2017M622144), Qingdao Postdoctoral Application Research Project, and Qingdao Young Scientist Applied Basic Research Fund (15-9-1-51-jch and 15-9-1-105-jch).

## Conflict of Interest

The authors declare that the research was conducted in the absence of any commercial or financial relationships that could be construed as a potential conflict of interest.
